# Building an Electronic Medical Record System Exchanged in FHIR Format and Its Visual Presentation

**DOI:** 10.3390/healthcare11172410

**Published:** 2023-08-28

**Authors:** Tz-Jie Liu, Hsu-Ting Lee, Fan Wu

**Affiliations:** 1 Department of Information Management, National Chung Cheng University, Chiayi 621301, Taiwan; 10544216@cycu.org.tw; 2 Center of Health Management, St. Martin De Porres Hospital, Chiayi 600044, Taiwan

**Keywords:** electronic health record, HL7, FHIR

## Abstract

Currently, the Taiwan Electronic Medical Record Exchange Center uses the Clinical Document Architecture (CDA) framework, which is based on the international medical standard. The CDA R2 standard, defined in 2005, is used for cross-institution retrieval of electronic medical records (Ministry of Health and Welfare, Information Department, 2021). However, CDA R2 only supports the exchange of clinical documents and is limited to the XML format. Due to the lack of a standardized framework for medical data exchange in Taiwan, different standards and specifications result in different data interface methods between systems, requiring customization for each system by healthcare institutions or the government. The inconsistency in data formats requires healthcare institutions and the government to spend more time on data parsing and mapping, resulting in slow integration of medical data. In this study, we simulated healthcare institutions using Fast Healthcare Interoperability Resources (FHIR) for medical information exchange and utilized the exchanged medical information to create a dynamic dashboard to assist healthcare professionals in making medical decisions. To ensure information security, we employed Hyper Text Transfer Protocol Secure (HTTPS) for secure transmission, which encrypts the transmitted medical record data using the Transport Layer Security (TLS) protocol, preventing deliberate interception and tampering of medical record data between the two systems. Finally, to test the load and performance of static and dynamic resources and web applications, we conducted a system performance evaluation using Apache JMeter. The results of this study demonstrate that replacing the gateway of the Electronic Medical Record Exchange Center with an FHIR server effectively reduces the time and cost spent by developers on data format conversion while also mitigating the information security risks associated with the previous VPN solution. Additionally, by utilizing dynamic charts, healthcare professionals are assisted in making medical decisions.

## 1. Introduction

Medical records are documents that store and record detailed information about a patient’s medical history, clinical findings, diagnostic results, pre- and post-operative care, and drug treatments [1]. According to the American Hospital Association (AHA), medical records are a compilation of data regarding a patient’s diagnosis, treatment, and care resulting from various general and specialized therapies. In China, medical records refer to the records made by medical personnel engaged in medical and healthcare work, documenting the various examinations, diagnoses, treatments, and care provided to patients during their medical business, as stipulated in Article 67 of the Medical Law [2].

In 2000, Taiwan’s Ministry of Health and Welfare launched an initiative to digitize medical records in healthcare institutions. Electronic medical records use digital technology to record patient information, and medical personnel can quickly access clinical data through electronic devices. This improves medical records’ completeness, accuracy, and real-time nature, where multiple individuals can access and operate them simultaneously [3]. Since 2011, the Ministry of Health and Welfare has established an Electronic Medical Record Exchange Center (EEC) to provide medical institutions with five types of medical records, including “outpatient medical records”, “outpatient medication”, “blood tests”, “imaging reports”, and “discharge summaries” [4]. This has enabled medical institutions to exchange and integrate medical records.

According to the Ministry of Health and Welfare’s statistics, 404 medical institutions, 5872 clinics, and 352 health centers in Taiwan have completed EEC interoperability and are using the Clinical Document Architecture (CDA) international medical standard to access electronic medical records across different institutions [5]. Taiwan’s Electronic Medical Record Exchange Center currently uses the CDA R2 standard defined in 2005. However, CDA R2 only supports the exchange of clinical documents and is limited to the XML format. Since Taiwan has not yet established standards for medical data exchange, different standards and specifications have resulted in different data interfaces between systems, requiring medical institutions and governments to customize interfaces for different systems. Each medical institution has its own way of expressing customized data. For example, some hospitals use “Female” or “Male” to represent men and women in database fields, while others use “1” or “2”. The inconsistent data formats make it challenging for medical institutions and governments to synthesize medical data [6], leading to slow data integration.

Fast Healthcare Interoperability Resources (FHIR) is an international health information exchange protocol proposed by the Health Level Seven International (HL7) organization in 2011. It promotes the exchange of healthcare information among healthcare providers, patients, caregivers, researchers, and anyone involved in the healthcare ecosystem. Compared with earlier HL7 medical information exchange standards, FHIR provides a unified standard specification that allows any healthcare provider to share information through the standard [7]. As of 2019, 84% of US medical institutions use FHIR technology, and the trend of using FHIR is expected to increase [8]. Taiwan also announced in 2020 that it would use the FHIR standard for its Electronic Medical Record Exchange Center [9].

FHIR is based on both HL7 v2 and HL7 v3 standards and is backward-compatible with all relevant past standards [10]. FHIR is a data model that primarily standardizes all data encountered in the entire healthcare system into a single data structure using a modular design [10]. Moreover, it is offered as a web service using the Representational State Transfer (REST) style through the Hyper Text Transfer Protocol (HTTP) for integration with other systems.

In addition to exchanging healthcare data, large amounts of data require more intuitive ways of being presented to find hidden information. Representing structured or unstructured data through visualization can make it easier to interpret meanings. Data visualization refers to the presentation of complex information as visual images. Using charts, healthcare personnel can quickly identify correlations between a patient’s diseases or abnormal values. This study also presents electronic medical record information through data visualization to provide users with an intuitive decision-making system to improve the quality of medical care.

**Figure 1:**
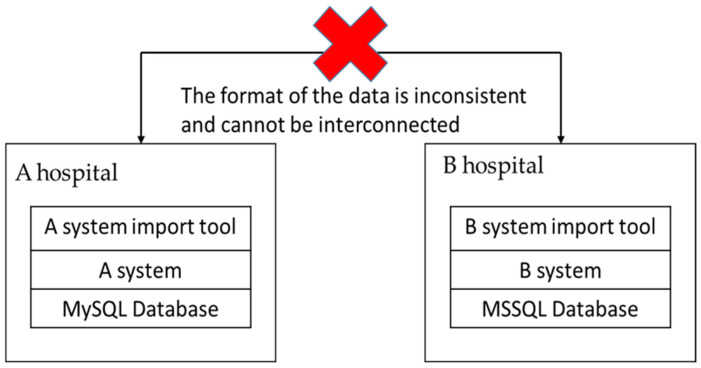
Difficulty of data exchange in medical institutions.

Presently, the information systems of Taiwanese medical institutions are typically provided by different information vendors, as shown in Figure 1. Vendors may each have different underlying data structures. A hospital’s medical information system may use MySQL, while another uses MS SQL. The lack of a unified format standard causes inconsistencies in data formatting, making it necessary for medical institutions to convert data formats when they have data exchange needs [10]. In the conversion process, the data structure, field rules, and interface used for integration require repeated discussion, leading to significant human and financial costs. According to statistics, it currently takes over two months on average to connect a medical institution’s system in Taiwan, and nearly every institution’s connection is a one-time project [6].

To solve the data exchange problem in the medical industry, HL7 proposed the medical information unified standard specification FHIR in 2011. Compared with the CDA standard, which only supports the Extensible Markup Language (XML) format, FHIR supports the XML format and other formats such as JSON (JavaScript Object Notation) and Turtle. Furthermore, FHIR adopts RESTful API as the data transmission method, which accesses data through HTTP and reduces the complexity of using the Simple Object Access Protocol (SOAP) by utilizing a simple and self-explanatory JSON syntax for data exchange. Users can manipulate data without having to learn SQL language. Regarding data interoperability, FHIR adopts the Cross-Enterprise Document Sharing (XDS) framework for electronic medical record exchange proposed by Integrating the Healthcare Enterprise (IHE). It also uses the Mobile Access to Health Documents (MHD) interoperability architecture that supports mobile application data exchange [11], enabling FHIR data to be transmitted across platforms and devices while addressing the low data interoperability problem under the CDA standard. Table 1 shows the differences between the CDA R2 standard and the FHIR standard currently used in Taiwan.

The purpose of this study was to develop an electronic medical record exchange system using the FHIR format, to simulate the process of exchanging electronic medical records, and to exchange data using a unified standard format without the need for complicated conversion processes, thus saving a significant amount of time and human resources in the interface. Additionally, the system visually displays the correlations between data through charts, allowing for the quick comprehension of disease information and trends, such as predicting the spread of infectious diseases and providing alerts to medical decision-makers.

## 2. Materials and Methods

### 2.1. EMR Format and Transmission Method

The Taiwan Electronic Medical Record Exchange Center (EEC) was established by the Ministry of Health and Welfare in 2011 to enable medical institutions to upload and access patient electronic medical records from different hospitals. The Taiwan electronic medical record exchange format currently uses the international standard HL7 CDA R2. All medical institutions must connect to the Electronic Medical Record Exchange Center through a virtual private network (VPN) provided by the Ministry of Health and Welfare. The information systems of each medical institution are not directly connected to the Electronic Medical Record Exchange Center but are connected to the center through an EMR gateway [12]. The EEC Gateway is a gateway host established in each hospital, and EEC can serve as a relay point through the gateway [13].

Medical institutions convert electronic medical records in their organizations into CDA standard files and store them on the EEC Gateway. When a medical institution needs access to a patient’s medical records from another medical institution, they must apply for the relevant electronic medical record data access through the patient’s health insurance card and the physician’s medical credential card and connect to the EEC platform. The EEC platform will notify the gateway storing the access data to return the complete data, and then, EEC will transmit the data back to the requesting gateway [13]. Although medical institutions must comply with the relevant regulations for producing and managing electronic medical records, there are no mandatory standards for the format and content of electronic medical records. Therefore, when medical institutions receive electronic medical record data with different standards, they must perform additional disassembly and field mapping to browse the data on their hospital information system (HIS).

Health Level Seven (HL7) is a non-profit standard development organization established in 1987 with the goal of developing standards for healthcare information systems (HIS) [14]. “Level Seven” corresponds to the computer interconnection standards set by the International Organization for Standardization (ISO) for open network architectures, specifically the seventh layer of the Open Systems Interconnection (OSI) model [15], which is the application layer. HL7 has developed standards for exchanging and managing electronic medical information for clinical and administrative purposes [16]. These standards allow medical information to be more effectively shared and exchanged to improve healthcare quality.

Fast Healthcare Interoperability Resources (FHIR) is a healthcare information exchange standard proposed by HL7 in 2011 [17]. FHIR is based on the web transfer standard HTTP RESTful protocol and uses the RESTful API as the data interface method. It supports the XML and JSON formats and can be used in various systems. FHIR is built on HL7 v2, v3, and CDA and is compatible with all relevant standards. Unlike complex CDA files, the FHIR data model consists of a series of resources that exchange clinical and non-clinical data in small unit sizes, such as basic patient data and financial information [17]. Therefore, developers can only request specific information.

In addition to defining the data exchange format, FHIR also defines interfaces. The FHIR API allows medical data to be quickly shared between two applications. FHIR supports five exchange frameworks [17,18]:

- RESTful API: used for FHIR applications or clients to request resource exchange from the server;
- Messaging: medical-related data are packaged into a set of FHIR resources for exchange using RESTful API or other message delivery technologies. This framework allows users to choose how to exchange medical information content according to their own architecture;
- Documents: medical-related data are packaged into content similar to HL7 CDA files;
- Services/SOA;
- Database/Persistent Storage.

The RESTful API is the healthcare industry’s most common and stable exchange framework for FHIR. The purpose of FHIR is to promote the interoperability of medical-related data. In the past, the complex architecture of v2 and v3 made planning to form clinical documents time-consuming. Additionally, a single exchange required the entire medical record data to be packaged into an XML file for transmission. In contrast, FHIR comprises data elements and formats; a medical record is divided into many resources, allowing the medical record reviewer to select the required medical record data. FHIR is compatible with past standards and does not require time-consuming conversion to a new standard format.

Representational State Transfer (REST) is an architectural style for networked systems proposed by Fielding [19]. REST is based on two Internet standards, the Hypertext Transfer Protocol (HTTP) and Uniform Resource Identifiers (URI). HTTP and URI define a common interface for the interaction of all resources on the Internet. HTTP is a network protocol divided into clients and servers. When a client sends a request to a server, the server accepts the request and then returns a response to the client.

HTTP defines several request methods, including the commonly used methods of GET, POST, PUT, PATCH, and DELETE. These correspond to the basic database operations of Create, Read, Update, and Delete. REST uses the HTTP protocol to express how resources are manipulated.

When a client sends a request, the objective is to obtain a resource, which may be a file or the result of a database query, among others. URI is how resources are identified and can be either a Uniform Resource Locator (URL) or a Uniform Resource Name (URN). A URL describes the network location of a resource, such as a web address, whereas a URN is a persistent, location-independent way to identify a resource using its name. Unlike a URL, which becomes unusable when a website’s content is moved to a new page, a URN allows users to locate the resource at any time.

### 2.2. System Design and Architecture

**Figure 2:**
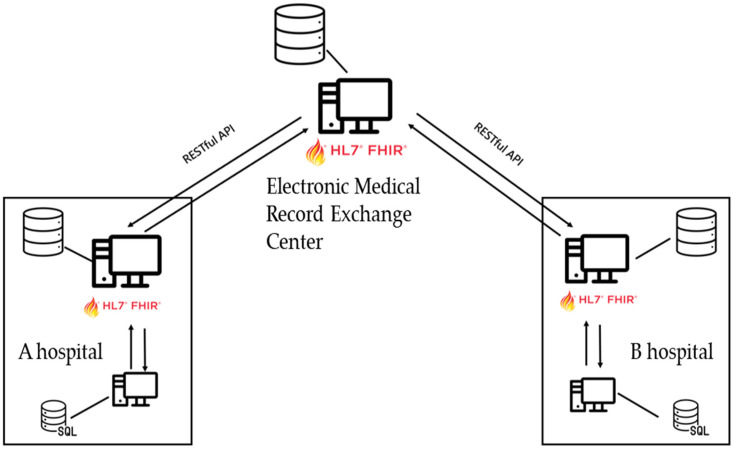
System architecture.

In the past, exchanging electronic medical records (EMRs) required storage and exchange through an EMR gateway. The present study simulated the exchange of EMRs between healthcare institutions through three Fast Healthcare Interoperability Resources (FHIR) servers using a centralized architecture, as shown in Figure 2. The FHIR servers can upload and download medical records through RESTful APIs. The middle server represents the Ministry of Health and Welfare’s EMR Exchange Center (EEC). In contrast, the other two servers represent two different healthcare institutions and simulate healthcare data exchange. When a healthcare institution has new medical record data, it can be directly uploaded to the institution’s server for temporary storage. The medical record data can be periodically uploaded to the exchange center server. When other institutions require access to the medical records, they can request access from the exchange center server that stores the medical record data.

Through this system, healthcare institutions can access patient medical record data and download it to their own systems or upload the medical record data to the exchange center server. Data can be accessed directly on HTTP through RESTful APIs without the need for data conversion. Medical record information can be presented visually, facilitating clinical decision-making by healthcare professionals.

#### 2.2.1. Installation of HAPI FHIR Server

Presently, there are many open-source servers available for users to choose from in the FHIR ecosystem. This study used the HL7 Application Programming Interface (HAPI) as the server. HAPI FHIR is an open-source project developed using the Java programming language and based on the HL7 FHIR standard. It was developed by the University Health Network (UHN), a Canadian healthcare research organization. It can fully achieve interoperability in healthcare and store health data that conforms to the FHIR format [20]. HAPI FHIR is widely used in several countries and is a user-friendly, convenient tool for building servers.

HAPI FHIR provides several ways of data deployment. In this study, the server was deployed using Docker, an open-source project that allows users to build and run any application anywhere. Unlike a virtual machine that needs to simulate an entire operating system through a hypervisor to run an application, Docker can run directly on the same host machine and execute multiple environments at once. Compared with a virtual machine, Docker is more lightweight and faster in execution. To simulate electronic medical record exchange, multiple servers must be deployed. Using virtual machines consumes more resources. Therefore, Docker was selected to deploy the server in this study.

Patient privacy is crucial. As such, this study used Hyper Text Transfer Protocol Secure (HTTPS) to encrypt medical record data via the HTTP protocol and Transport Layer Security (TLS) during transmission. This measure prevents malicious interception and tampering of medical record data transmitted between two systems.

Node.js is a runtime environment designed to execute JavaScript on the server side. It is based on JavaScript and provides a unified development environment for both front-end and back-end languages, improving efficiency and convenience. This study selected Node.js as the back-end language, and the Express framework was used as the back-end framework. Express is a framework based on the Node.js web server, simplifying the HTTP service in Node.js. For example, when Node.js calls the GET method, it needs to check the request’s URI and method, which is called routing. Writing multiple lines of code is required to solve the problem of being time-consuming. However, using Express, this can be achieved in just a few lines of code, making it easier for Node.js to write web applications.

#### 2.2.2. Electronic Medical Record Exchange Process

The proposed system in this study can exchange patient data and various test results as resources, using the Patient Resource and Observation Resource for subsequent exchange and information presentation. When a user wishes to view patient data stored on the FHIR server, a request is made to the FHIR URL via the HTTP GET method, and the RESTful API can directly operate the FHIR server to obtain all patient data.

Each piece of data in the FHIR server has a specific serial number for identification. When the organization’s FHIR server uploads patient data to the exchange center server, it first captures the URLs of the two servers. Then, it obtains the resources stored on the organization’s server through the organization URL and captures the patient’s identification number to verify whether the exchange center server has this patient’s data. If there is a duplicate identification number problem, the patient’s date of birth is used as the judgment basis.

**Figure 3:**
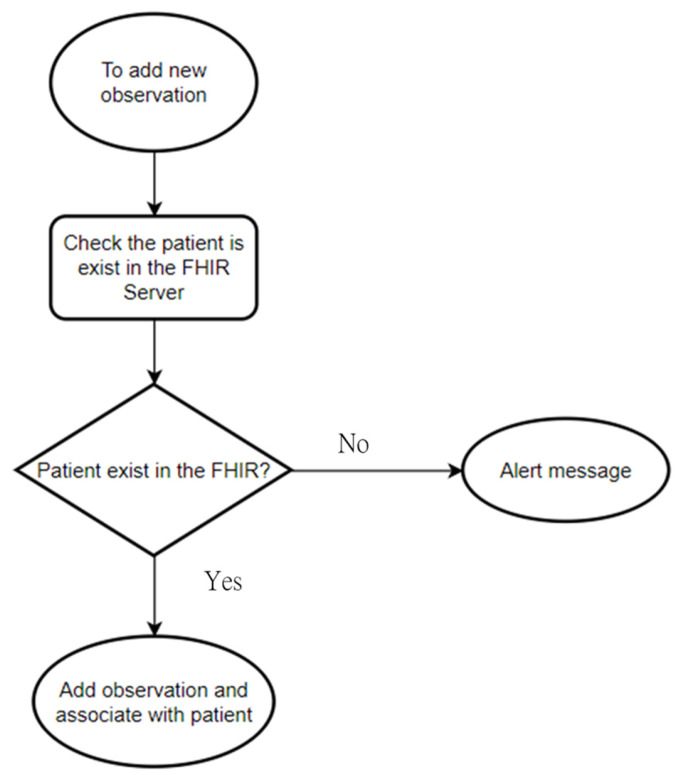
New test value flow chart.

When adding new test results, the system first captures the serial number of the associated patient test result in the server as the basis for addition. As shown in the Figure 3 flowchart, when a user uploads a test result, the system first captures the subject within the observation. It checks the patient’s identification number through the content of the serial number in the reference and judges whether the patient exists based on the patient’s identification number.

#### 2.2.3. Graphical Analysis of Medical Records

**Figure 4:**
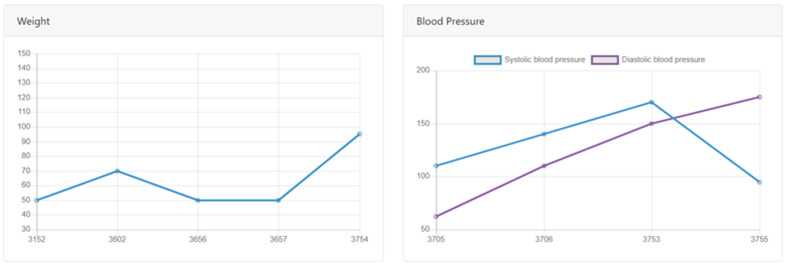
Diagram of medical records.

This study presents a dashboard using the Ministry of Health and Welfare’s public data on dengue fever cases by region, age group, and gender since 2003, as well as weight and blood pressure FHIR data received and uploaded by the system. After obtaining medical data, D3.js and Chart.js present a visualized dashboard interface for medical decision-makers. The interface is divided into individual medical record data and dengue fever data across Taiwan, as shown in Figure 4. Medical staff can quickly view all the examinations and test values that the patient has undergone during their visit and can identify physiological trends to determine the appropriate diagnosis and treatment.

### 2.3. Development Environment and Software Specifications

This system was tested and evaluated on a machine with a Windows 10 operating system. The memory was 32GB RAM and the processor was i7-8700 CPU. Using HTML, CSS, and JavaScript, the dashboard interface was written using JavaScript libraries D3.js and Chart.js.

### 2.4. System Evaluation

This study compared the content and file size of medical data transmitted under the FHIR and CDA R2 standards to explain why this system uses the FHIR format for data exchange. Apache JMeter tested the system’s response time and throughput to evaluate its performance and to provide an explanation.

#### File Structure and Size Comparison

**Figure 5:**
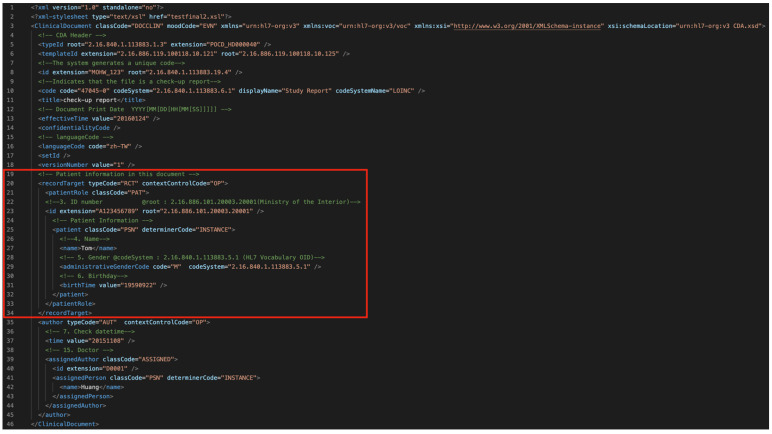
CDA file line count.

**Figure 6:**
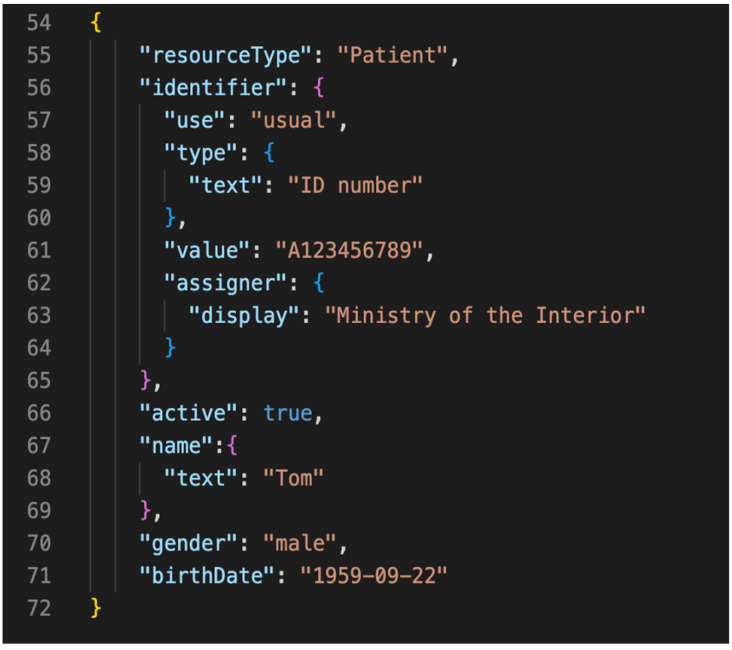
FHIR file line count.

According to the study by Wenxin Zheng [21], which transformed a single Ministry of Health and Welfare CDA data into FHIR format, FHIR files comprise template components that allow users to select desired data. In contrast, CDA data are large in size and have fixed fields. Compared with CDA, FHIR data are significantly smaller, and using CDA for transmitting smaller medical data files would result in additional data transmission burdens. As shown in Figure 5 and Figure 6, using a health check report as an example, if we only need patient data in the red box in Figure 5, CDA would need to package and transmit the entire data, including all test values. In contrast, FHIR would only transmit the selected patient resource directly.

### 2.5. System Performance Verification

Performance is crucial in a system, especially when there is a large amount of health data concerned. This study used two evaluation methods to assess system performance. The first method evaluated system response time. Time To Last Byte (TTLB) refers to the time it takes for the entire process, from when a client sends an HTTP request to when they receives a response from the server. Slow system response time will render the response impractical for actual use. A highly efficient system, including those used in clinical settings, should ideally have a response time of less than 0.1 s and an acceptable response time of less than 2 s. Users may leave the application if the response time exceeds 10 s. The second evaluation method examined the system’s throughput, which refers to the number of requests a system can process in a specific period as the number of clients increases.

Apache JMeter was used in this study to test the response time and stress of the system. Apache JMeter 5.4.3 is an open-source software written in Java and can be used to test the load and performance of both static and dynamic resources and web applications. The performance evaluation method in this study involved sending two types of requests to the system: GET requests to obtain medical information and POST requests to send data. The response time efficiency of the two types of requests was tested to determine whether the system’s throughput will be significantly affected as the number of users simultaneously requesting increases. Finally, the application performance index (Apdex) was evaluated, which is a standard for measuring application performance satisfaction and ranges from 0 to 1, where 1 represents all users who are satisfied and 0 represents no user satisfaction.

### 2.6. Apdex

Application performance index (Apdex) can evaluate users’ satisfaction with this system. When users use this system to access medical data, the longer the waiting time, the lower the user satisfaction will be. The range of Apdex is between 0 and 1, and the closer to 1, the higher the satisfaction. The calculation formula of Apdex is as follows [22]:

$$Apdex=\frac{SatisfiedCount+\left(ToleratingCount*0.5\right)+(FrustratedCount*0)}{TotalSamples}$$

Among them, Satisfied Count is the number of satisfied samples, Toleration Count is the number of samples that can tolerate the threshold, and Frustration Count is the number of samples that are restless. Table 2 shows the relationship between value and satisfaction, the lower the value, the lower the satisfaction:

## 3. Results

Three tests were performed to compare the effect of the number of requests on performance. Each test was scheduled to have the designated number of users make requests within one minute, with a fixed request content. The first evaluation involved 100 users making 100 requests each, resulting in 10,000 requests sent to the system within one minute. The second evaluation involved 200 users making 100 requests each, resulting in 20,000 requests sent to the system within one minute. Finally, the third evaluation involved 400 users making 100 requests each, resulting in 40,000 requests sent to the system within one minute. The evaluation results and analysis are presented below.

Table 3 summarizes the system performance when simulating 100, 200, and 400 users, each making 100 GET requests. Table 4 summarizes the system performance when simulating 100, 200, and 400 users, each making 100 POST requests. The “Samples” represent the total number of requests sent within one minute during the test. For example, in the first test, where 100 users each made 100 requests, the “Samples” column displayed 10,000. “Average” represents the average response time for a single request. “Median” represents the response time for 50% of users. The “90% Line” represents the response time for 90% of users. “Min” and “Max” represent the minimum and maximum response times, respectively. “Error” represents the percentage of requests that encountered errors during the test. “Throughput” represents the number of requests that can be completed per second.

In the simulation of 10,000 GET requests, the average time was 87.71 milliseconds (i.e., 0.08771 s), with a 90% user response time of 145 milliseconds (i.e., 0.145 s) and an error rate of 0.00%. In the simulation of 20,000 requests, the average time was 179.46 milliseconds (i.e., 0.17946 s), with a 90% user response time of 284 milliseconds (i.e., 0.284 s), and an error rate of 0.01%. Finally, in the simulation of 40,000 requests, the average time was 119.55 milliseconds (i.e., 0.11955 s), with a 90% user response time of 194 milliseconds (i.e., 0.194 s), and an error rate of 0.00%. For the GET performance evaluation, the average response time for all three tests was within an acceptable range, with user response times within 2 s. However, the maximum response time for 20,000 requests was 4710 milliseconds (i.e., 4.71 s), possibly due to a nonresponsive error during testing. System throughput increased as client requests increased, with a throughput of 133.28 requests per second for 10,000 requests, 176.89 requests per second for 20,000 requests, and 194.14 requests per second for 40,000 requests.

In the simulation of 10,000 POST requests, the average time was 95.17 milliseconds (i.e., 0.09517 s), with a 90% user response time of 157 milliseconds (i.e., 0.157 s) and an error rate of 0.00%. In the simulation of 20,000 requests, the average time was 398.73 milliseconds (i.e., 0.39873 s), with a 90% user response time of 694 milliseconds (i.e., 0.694 s) and an error rate of 0.00%. Finally, in the simulation of 40,000 requests, the average time was 1318.84 milliseconds (i.e., 1.31984 s), with a 90% user response time of 1814 milliseconds (i.e., 1.814 s) and an error rate of 0.00%. In the POST performance evaluation, although the response time increased gradually with the increase in users, the average response times of all three tests were within an acceptable range, with user response times within 2 s. However, the maximum response time for 40,000 requests was 5013 milliseconds (i.e., 5.013 s), possibly due to insufficient performance in the system’s 32GB memory environment. The system throughput increased as client requests increased, with a throughput of 133.27 requests per second for 10,000 requests, 176.91 requests per second for 20,000 requests, and 195.09 requests per second for 40,000 requests.

**Figure 7:**
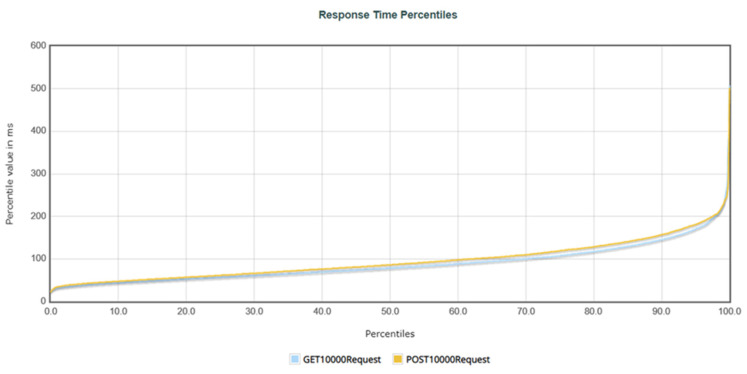
The response time percentages for 10,000 requests.

Response time percentiles refer to the percentage of requests with response times within an acceptable range compared with all requests used to measure the stability of a system. In Figure 7, the *X*-axis represents the percentage of requests, while the *Y*-axis represents the response time. Moreover, the response percentiles for 10,000 requests are sent to the system, indicating that the curves for POST and GET requests were similar. When the request percentage reaches 99%, both types of requests have response times within 500 milliseconds (0.5 s), and the curves are smooth, indicating that the system is very stable when 10,000 requests were sent to the server.

**Figure 8:**
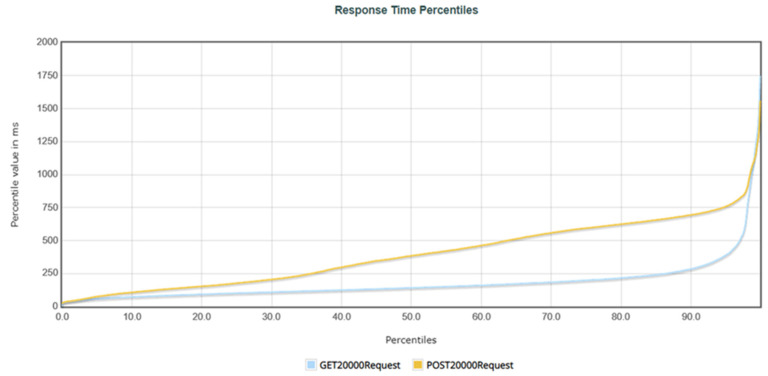
The response time percentages for 20,000 requests.

Figure 8 shows the response percentiles for 20,000 requests sent to the system. When the request percentage reached 99%, the response time for POST requests was 1102 milliseconds (1.102 s), and the response time for GET requests was within 1092 milliseconds (1.092 s). Although the response time for POST requests was longer than that for GET requests, the curve remained stable, indicating that the system remained stable during the 20,000 request tests.

**Figure 9:**
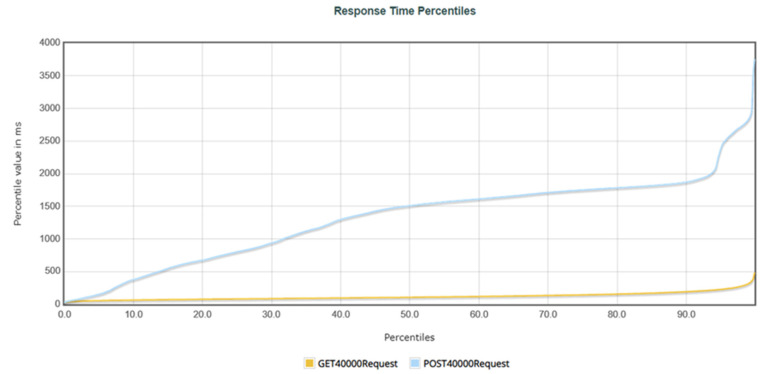
The response time percentages for 40,000 requests.

Figure 9 shows the response percentiles for 40,000 requests sent to the system. When the request percentage reached 99%, the response time for POST requests was within 2827 milliseconds (2.827 s), while the response time for GET requests was within 315 milliseconds (0.315 s). The curve for POST requests gradually increased as the number of requests increased to 40,000, while the curve for GET requests remained relatively flat. As the system has 32 GB of memory, a large number of users uploading data can cause performance problems, resulting in longer response times for POST requests. However, both curves show no significant fluctuations, indicating system stability during the 40,000 request tests.

**Figure 10:**
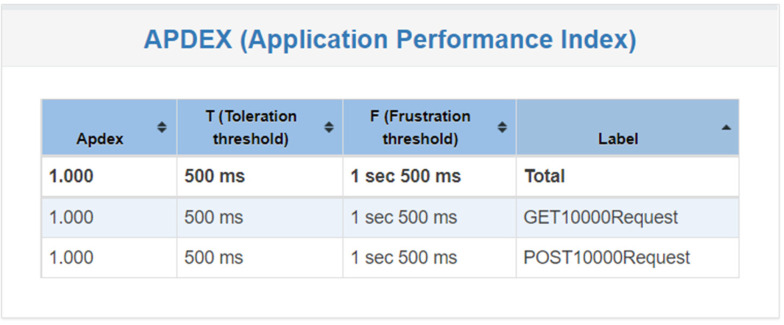
System satisfaction of 10,000 requests.

Figure 10 shows that, when the system is tested 10,000 times, the user satisfaction is 1, which means that the user experience is very good when there are 100 users at the same time and each user operates 100 times.

**Figure 11:**
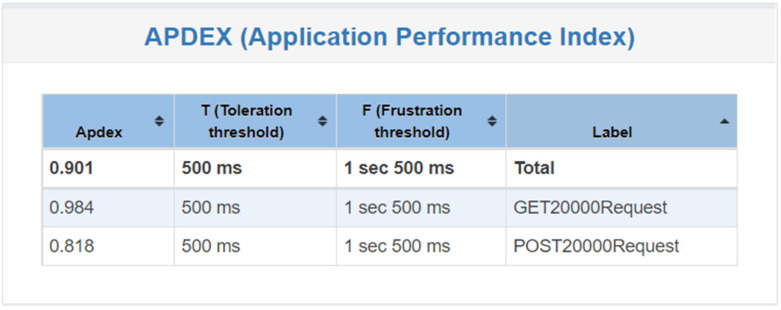
System satisfaction of 20,000 requests.

Figure 11 shows that when the system is tested 20,000 times, the user satisfaction falls between 0.9 and 0.8, indicating that the user experience is good when there are 200 users at the same time and each user operates 100 times.

**Figure 12:**
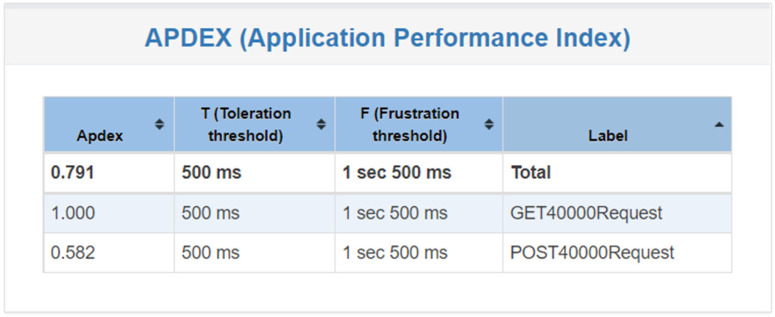
System satisfaction of 40,000 requests.

Finally, Figure 12 shows that when the system has undergone 40,000 tests, the user’s satisfaction rate is 1 when obtaining patient or medical record data, but the satisfaction level is relatively low when uploading or downloading data, which means that the user has 400 users at the same time. Or, when each user operates 100 times, the user experience is poor. In the previous section, it can be found that when POSTing 40,000 pieces of data, the required response time is 2.8 s, mainly because the system has insufficient memory and there are 40,000 pieces of requests at the same time. The time performance is poor, so increasing the memory space can solve this problem.

## 4. Discussion

This study developed an electronic medical record exchange system using FHIR as the exchange format. The development of an electronic medical record exchange system using Fast Healthcare Interoperability Resources (FHIR) as the exchange format has significant implications for the field of hospital information transmission in many aspects. Firstly, the study’s findings can promote interoperability among different hospital systems. Since FHIR is designed to facilitate the exchange of healthcare information between different software applications and systems, by implementing an electronic medical record exchange system based on FHIR, hospitals can overcome the challenges of disparate or heterogeneous systems and enable seamless communication and sharing of patient data. This promotes better continuity of care and improves the overall efficiency of information transmission within and between healthcare facilities. Secondly, the use of FHIR as the exchange format can enhance data standardization and compatibility of different hospital information systems. FHIR provides a standardized and structured approach to representing and exchanging healthcare data, ensuring that information is easily understood and interpreted across systems. This enables hospitals to transmit and receive patient records, test results, and other vital information in a consistent and standardized format, reducing errors, misinterpretations, and data inconsistencies. Additionally, the study’s implications include improved efficiency and workflow optimization. The development of an electronic medical record exchange system based on FHIR can automate the transmission and retrieval of information, reducing the reliance on manual processes. Clearly, this new FHIR transmission can save time, reduce administrative burdens, and enhance the overall productivity of healthcare professionals involved in information transmission. Moreover, by leveraging FHIR’s capabilities, hospitals can enhance data security and patient privacy. FHIR supports advanced security features and protocols, enabling secure access and transfer of sensitive patient information. The study’s implications can help hospitals implement robust security measures and ensure compliance with privacy regulations, thereby safeguarding patient data throughout the information transmission process. In summary, the implications of the study for the field of hospital information transmission include improved interoperability, standardized data exchange, enhanced efficiency, and optimized data security. Implementing an electronic medical record exchange system using FHIR can facilitate seamless communication, improve patient care quality, and drive advancements in the management and transmission of healthcare information within hospital settings. The research results can provide a reference for Taiwan’s future electronic medical record exchange architecture. However, this study simulates the exchange of electronic medical records (EMR) using a system in FHIR format, lacking a research setting for the practical implementation challenges and outcomes of such systems in real-world healthcare. Future work can be more practical and in-depth research that can be conducted in five directions, namely, system integration, privacy and security, usability and user training, testing and validation, and scalability of healthcare delivery and outcomes.

## 5. Conclusions

Taiwan’s electronic medical record exchange format is primarily based on the CDA R2 standard established in 2005 and only supports the XML format. This medical data transmission method requires converting medical records within the medical institution to the government-approved medical record format before uploading them to the gateway of the Electronic Medical Record Exchange Center for storage and use. However, the government has yet to establish a regulated format for electronic medical records, resulting in the need for uploading institutions to spend time on conversion and for medical institutions accessing the records to convert the received data to conform to their own custom format. Taiwan has been working on converting the CDA standard to the FHIR standard in recent years. However, the massive amount of medical information cannot be converted overnight. Therefore, the Ministry of Health and Welfare has taken COVID-19 as the first step in converting to the FHIR architecture, establishing a vaccine passport using FHIR as the medical information standard. Through the FHIR format, Taiwan can directly exchange medical information with foreign countries.

## Figures and Tables

**Table 1:** Differences between the current CDA R2 and FHIR standards in Taiwan.

	CDA R2 in Taiwan	FHIR	Reasons for FHIR’s Success
Resource of Data Format	XML	XML, JSON, Turtle	Supports multiple formats with high flexibility
Interoperability support	Similar to IHE-XDS	IHE-XDS, IHE-MHD	Supports mobile devices and resource-limited systems, such as smartphones and IoT devices
Data access	Custom API	RESTful API	High consistency with mainstream web technologies
Specification changes	EEC developed it with 4 to 13 pages per interoperability specification presented in Word documents	The official website has a registration for the implementation guide, which is presented as a website and includes data format, fields, sample files, etc.	Relatively comprehensive and easy to read
Data validation	Requires inquiries to EEC personnel to obtain relevant testing files for online testing	Testing can be performed on an open test or a self-built FHIR server	Shortens the time for going live
Specification development	Designed by different people with relatively large differences	Designed by different people but with smaller differences	Specification development is more standardized
Data types, fields, and structures	Based on v3 and RIM, which are relatively abstract	Based on FHIR	Relatively easy to understand

**Table 2:** Apdex reporting rules.

Rating	Apdex Value Range
Excellent	0.94–1.00
Good	0.85–0.93
Fair	0.70–0.84
Poor	0.50–0.69
Unacceptable	0.00–0.49

**Table 3:** GET report.

Samples	Average	Median	90% Line	Min	Max	Error	Throughput
10,000	87.71	78	145	24	508	0.00%	133.28/s
20,000	179.46	141	284	1	4710	0.01%	176.89/s
40,000	119.55	102	194	22	1496	0.00%	194.14/s

**Table 4:** POST report.

Samples	Average	Median	90% Line	Min	Max	Error	Throughput
10,000	95.17	86	157	21	502	0.00%	133.27/s
20,000	398.73	141	694	21	2739	0.00%	176.91/s
40,000	1319.84	1535	1814	21	5013	0.00%	195.09/s

## Data Availability

The data are available upon request.
